# Elicited emotion: effects of inoculation of an art style on emotionally strong images

**DOI:** 10.1007/s00221-025-07030-x

**Published:** 2025-03-12

**Authors:** Amparo Caceres Gutierrez, Julián Tejada, Enrique García Fernández-Abascal

**Affiliations:** 1 National University of Distance Education UNED, C. de Bravo Murillo, 38, Chamberí, 28015 Madrid, Spain; 2 Department of Psychology, Universidade Federal de Sergipe, Aracaju, Brazil; 3 Department of Basic Psychology II, National University of Distance Education, Office 2.38, Faculty of Psychology, UNED, Juan del Rosal 10, 28040 Madrid, Spain; 4 Faculty of Psychology, National University of Distance Education, UNED, C. de Bravo Murillo, 38, Chamberí, 28015 Madrid, Spain

**Keywords:** Artistic filter, Emotional response mitigation, Convolutional neural network

## Abstract

The objective of this research is to study how the application of the Convolutional Neural Network (CNN) artistic filter can be an alternative to mitigate the emotional response to photographs with strong emotional content published in Internet news. Van Gogh’s artistic style was extracted through a CNN and inoculated with 64 IAPS images chosen to cover the entire emotional space. 140 university students of both sexes (70 men and 70 women) with an average age of 22 years, evaluated 128 stimuli, 64 original and 64 digitally inoculated, giving the appearance that they were painted with the artistic style of Van Gogh. For the evaluation of the stimuli, four groups were established under the conditions: 1 high arousal—positive valence, 2 negative valence—low arousal, 3 high arousal—negative valence and 4, low arousal, positive valence. The original images (OI) tended to produce less pleasant effects, while the images inoculated with filters made with CNN provoked reactions with a tendency to calm. The most significant changes in the emotional states are observed in the valence, the stimuli with the inoculation of the artistic style produces alterations with a tendency to pleasant effects. The averages of the comparisons of the dimensions valence and arousal of the OI and the CNN allow to show that there are differences in the emotional states, the results can permit the development of a methodology that, based on the inoculation of the artistic style of original paintings through CNN in emotionally strong images, a new image is created that replaces the strong images published in the Internet news.

## Introduction

The image is a very effective tool to transmit emotions online, the increasing ease of manipulating and distributing images through mobile devices and social networks allows users to share their ideas, thoughts, emotions and experiences with other people generating an emotional response (Lang 1979; Joshi et al., 2011; Jou et al. 2015; You et al. 2015; You et al. 2016; Wang 2020; Shahzad and Alhoori 2021; Wan et al. 2022). Studies show that viewers spend more time on websites with manipulated or fake images. These images, often accompanying online news, can be emotionally distressing and misleading. Because viewers are often unaware that images can be altered, they are more vulnerable to misinformation, which can influence their opinions and actions. (Zillmann et al., 1999; Fahmy et al. 2014; Stumpf, 2017; Kasra et al. 2018; Shen et al. 2019; Knobloch et al. 2003; Vraga et al. 2020; Vieweg 2010; Mohd Shariff et al. 2014; Guidry et al. 2015; Lewandowsky et al. 2012; Lazer et al. 2018; Vázquez- Herrero, 2019).

Visually-focused social media platforms attract long visits, but they also expose users to a higher risk of encountering false information presented through images. (Vieweg 2010; Mohd Shariff et al. 2014; Guidry et al. 2015; Lewandowsky et al. 2012; Lazer et al. 2018; Vázquez-Herrero 2019).

Images in online news serve a dual purpose: to document facts and to evoke emotions. Strong images are particularly effective at stirring emotions, often more so than other forms of media. Therefore, the emotional content is the most important attribute of a news photograph (Sharkey 1993; Graham 2000). Negative images (unpleasant-threatening) have a strong presence in online journalism, the emphasis on visual content, design and aesthetics favor the emotional image, which stimulates user participation, capturing greater attention and impact (Rivers and Mathews 1988; Zillmann et al. 1999; Pavlik 2001; Knobloch et al. 2003; Rössler et al. 2011; Stumpf, 2017; Keib et al. 2018; Li and Xie 2020; Vraga et al. 2020).

The study of the criteria for the selection and impact of press photography (display of strong emotions, broad colours, vivid images) and the effect on the recipients (level of memory, evaluation of the emotional impact of the image and duration of observation) shows the influence on attention to attract and perceive the image (Demarmels 2007; Rössler et al. 2011).

The study on understanding the meaning of photo content shows that photos with faces are more likely to be liked and commented on by Instagram users (Bakhshi et al. 2015). News that contains images attracts more attention and encourages more user engagement than text-only news.,The emotional impact of these images depends on the careful selection of the subject matter and the capturing of a particularly meaningful moment in time,. These are the key elements that make a news photograph powerful (Chiorean 2016; Rivers and Mathews 1988; Keib et al. 2018; Li and Xie 2020).

Fake news spreads faster, further and more often than real news. When selecting journalistic stories, users click more often on certain topics, such as terrorism, natural disasters, or urban legends, specially if these topics contain emotional words classified as trust, anticipation or anger, which can evoke fear, disgust and surprise (Winter and Krämer 2014; Vosoughi et al. 2018; Pröllochs et al. 2021).

In order to detect and respond to the spread of strong photographs in digital news, content moderators and algorithms to monitor and control the dissemination of impactful photographs are used. (Seering et al. 2019; Roberts 2019; Gerrard 2020). Content moderators are people responsible for filtering, flagging, or censoring the content of inappropriate material and potentially dangerous information (Freire et al. 2022). Elements that define the removal criteria include the most extreme end of the graphic content spectrum, such as child exploitation, animal cruelty, suicide and murder. However, despite their best efforts, they cannot prevent all strong images from being filtered (Gillespie et al. 2020).

Algorithmic moderation systems are management mechanisms that structure participation in an online community to facilitate cooperation, prevent abuse, and remove illegal content quickly and effectively (Grimmelmann 2015). The goal of algorithmic moderation is to identify, match, predict, or classify some strong content, such as an image, based on its exact properties or general characteristics. Automated algorithmic moderation tools that platforms use to police content across a wide range of subject areas of scale photography, including terrorism, graphic violence, and toxicity, are applied when scale issues make manual moderation impossible (Gorwa et al. 2020).

Much of the correction of misinformation in online news focuses on the textual content. However, some studies indicate that the use of the Picture-O-Meter barometer allows evaluating whether a photograph is authentic or not, a forensic label is placed in photography to influence the perception of credibility of images (Shen et al. 2019, 2021). To ensure the authenticity of digital images and mitigate misinformation, different watermarking techniques have been developed using algorithms; bit it is difficult to achieve a watermarking system that is both robust and secure (Wan et al. 2022).

## Emotion and art style

Emotions are psychophysiological processes that help the organism to adapt to the environment and achieve its goals, they have an adaptive and evolutionary character, since they prepare the organism to emit a behavior in response to a demand from the environment, for example, fear prepares for a fight/flight response, anger for protection or joy for approach (Lang 1995, 2010; Eisenberg 2000). The bioinformational model of emotions defines emotion as a state of predisposition to action based on the activation of one of the two primary motivational systems, appetitive or defensive, which is associated with approach/consummation or escape/avoidance behaviors (Lang 1995).

From this perspective, emotions are dispositions to action that arise in response to stimuli that are meaningful to the individual and are generated in a triple response system: cognitive/experiential/subjective; motor/behavioral/expressive, neurophysiological or cognitive associated with the context in which they develop and the evaluation is carried out from three dimensions, valence, arousal and dominance (Bradley 2009; Lang 1995, 2010; Bradley and Lang 2007).

Emotional experience can be understood through three key dimensions: valence, arousal, and dominance. Valence refers to the positivity or negativity of an emotion, ranging from pleasant to unpleasant. Arousal describes the intensity or activation level of the emotion, ranging from excitement to calmness. Dominance relates to the sense of control a person feels over their emotional response, influencing whether they approach or avoid a situation. (Bradley and Lang 2000; Vila et al. 2001; Coan and Allen 2007).

Emotions and art are intimately linked, from antiquity to the present day, theories of aesthetics have emphasized the role of art in the evocation, configuration and modification of emotions and are at the basis of aesthetic evaluations of objects and works of art (Leder et al. 2004; Silvia 2009; Schindler et al. 2017; Pelowski et al. 2016; Fingerhut and Prinz 2018). Artworks can evoke a wide variety of emotional responses, such as calmness, dynamism, agitation, happiness, anger, fear, disgust, sadness, shock, and erotic desire, these emotions can arise when a person perceives and evaluates a stimulus for its aesthetic appeal (Zhao et al. 2017; Cupchik 2015; Lu et al. 2016).

In painting, humans have mastered the skill of creating unique visual experiences by composing a complex interplay between the content and artistic style of a work of art (Gatys et al. 2015a, b). Artistic style is a multidimensional variable related to the individuality of the artist, the similarity of the aesthetic composition, the space and the harmonization between the particularity of the artist, the trend of the time and society (Jr et al. 2008; Cupchik 2015).

A painter’s unique artistic style is defined by the consistent use of elements like dominant colors, size, line, lighting, texture, shape, and space, as well as the way they utilize their materials. This combination creates the work’s content and shapes the viewer’s aesthetic experience (Zhao et al. 2021). It is common for artists to use the environment as a basis to create works that reflect their emotions and artistic style (Datta et al. 2006; Karayev et al. 2014a, b; Peng 2017; Huang and Belongie 2017; Li et al. 2017; Ionescu et al. 2022; Liao and Huang 2022).

The viewer’s initial encounter with a work of art involves a rapid analysis of its stylistic characteristics. This assessment creates a basic impression of the work’s structure and meaning, stimulating the viewer to interpret its various elements and relate them to their own past experiences in a meaningful way. (Leder et al. 2004; Van Paasschen et al. 2015).

## Digital filters and CNN

The use of computer technology to apply digital filters to images posted on the internet has become a way for people to express and communicate affection and emotion, filtered photos on social networks are more likely to be viewed and commented on (Melo and Gratch 2009; Bakhshi et al. 2015). Digital filters are tools that allow you to inoculate color, adjust shadow, texture, light exposure or simulate a change in focus of an image, create special effects such as stylizing, saturating or aging a photo by changing the emotional salience of the image, changing the emotional state of the user (Bakhshi et al. 2015; Chandler and Livingston 2016; Murrin 2017).

The filter significantly changes the look of photography by providing users with convenient and fun ways to distance their images from reality, producing an artistic experience (Trope and Liberman 2010). Studies related to the psychology of the digital filter examine how photographic filters can affect perception and increase viewers’ levels of interpretation by perceiving images in a more abstract and interpretive way (Peng and Iii 2018).

Dark filters, such as black or monotone, are often associated with negative emotions like anger, fear, sadness, and disgust. Conversely, light filters tend to be linked to positive emotions such as surprise, anticipation, trust, and joy. Images with high valence (more positive) are generally more engaging and captivating to viewers, while those with low valence (more negative) can convey feelings of negativity (Murrin 2017).

When predicting the emotional content of images based on valence and arousal, researchers found that certain visual features offer valuable clues. Texture characteristics were strongly associated with calmness. Roundness-angularity and simplicity-complexity were found to correlate equally with both valence and arousal, particularly when comparing images with strong emotional content to neutral images (Lu et al. 2016).

Research on the nostalgic consumption of photography among young people reveals that Instagram consumers give value to their experience of everyday life through the use of digital filters with nostalgic effects applied to contemporary photographs. For the group of participants evaluated, the photographs that look older are perceived as more narrative, because they have more power to tell stories (Morlot 2013).

Studies on the motivation of users to filter photos on the Internet, have shown that they prefer to focus specifically on algorithms that are trendy, popular, aesthetically appealing, aimed at social interaction, or the imagination of the audience, or that control the quality of the photo, correcting errors such as lighting, shadows and sharpness (Hutto et al. 2013; Lavrence and Cambre 2020).

This is how techniques to automate the coding of visual content, through filters,make it possible to understand diffusion patterns and the different characteristics of images to interpret emotional content (Datta et al. 2006; Sermanet et al. 2014; Yuan et al. 2013; Krizhevsky et al. 2014; You et al. 2017; Joo Steinert-Threlkeld 2018; Zhao et al. 2021).

In experimental research, automatic classification algorithms were used to evaluate the aesthetic quality of photographs found on websites. The algorithms extracted features such as light exposure, saturation, and hue. Additionally, the rule of thirds was applied, dividing each image into nine equal parts to identify key points of interest. The results indicated a correlation between these extracted features and the emotions evoked by the images. (Machajdik and Hanbury 2010). Another study used automatic classifiers to discriminate between aesthetically pleasing and unpleasant images, by extracting visual features (familiarity, texture, size and aspect ratio, composition, depth of field, shape convexity) (Datta et al. 2006; Joshi et al. 2011).

Research on how artistic principles influence the emotions conveyed by images has employed various methods. For example, Zhao et al. (2017) used an algorithm to extract artistic style characteristics to classify and evaluate image emotions. In another study, Johnson et al. (2007) analyzed Van Gogh’s post-impressionist style through automatic stroke extraction and color management. Their results demonstrated the expressive effects of his paintings, revealing how color reflected the content, his interactions with other artists, and his emotions.

The aesthetics and emotional values of images are related to their meaning, the extraction of image characteristics such as color, texture and shape allows the recognition of emotions in images and art (Joshi et al. 2011). Likewise, psychological studies show that the aesthetic response of an image depends on dimensions such as composition, color, spatial organization, emphasis, movement or depth (Axelsson 2007; Freeman 2007; Peters 2007).

Studies relating to experimental aesthetics establish that various aspects of color perception play a relevant role in preference judgments, more saturated colors are correlated with positive ratings for valence, images with high valence values will be more interesting, thus captivating the viewer, while images with low values transmit emotions of negativity (Datta et al. 2006; Palmer and Schloss 2010; Mallon et al. 2014).

In this regard, the concept of psychological studies and art theory are used to extract characteristics from images, thus allowing the recognition of a variety of emotions in these images (Machajdik and Hanbury 2010). In the arts, emotionally negative objects can be judged positively, the results relating to emotional experiences (joy, anger, disgust, fear, sadness and shame) and aesthetic judgments show that the aesthetic evaluation of emotionally negative objects can be evaluated in a positive way (Gerger et al. 2014).

Researches related to visual emotions expressed in images establish that through different combinations of aspects of the artistic style of the image such as balance, gradation, movement, rhythm and proportion, emotions can be evoked. symmetrical and harmonious images tend to evoke positive emotions, while images that present strong color contrast can evoke negative emotions (hatred, anguish, anxiety) (Collingwood 1993; Zhao et al. 2017; Ruskan 2021).

Computational methods permit to record in the perceived images, aspects applied to aesthetics and the evocation of emotions. These methods are based on the processing of points or pixels to alter the color and brightness of each pixel (Stork 2009; Peng 2017).One of the methods that stands out are the Convolutional Neural Networks (CNNs), which consists of multiple layers of filters of small computing units that hierarchically process visual information. Each layer of units is understood as a collection of image filters, where each one extracts a certain feature from the input and output image. They are the so-called feature maps, which are versions of the input image with different filters (Nolan 2019).

One of the methods used in the training of the CNN is to decode an original photograph by transferring it to an artistic style of a work of art, the neural algorithm used for the synthesis of the artistic style is an artificial system of neural networks that allow arbitrary separation and recombination the content and style of images, to create high-quality artistic images, controlling the design of the image in an abstract style from the content of a real photograph and the style representation of a work of art (Gatys et al. 2015a, b; Zhao et al. 2018).

**Fig. 1:**
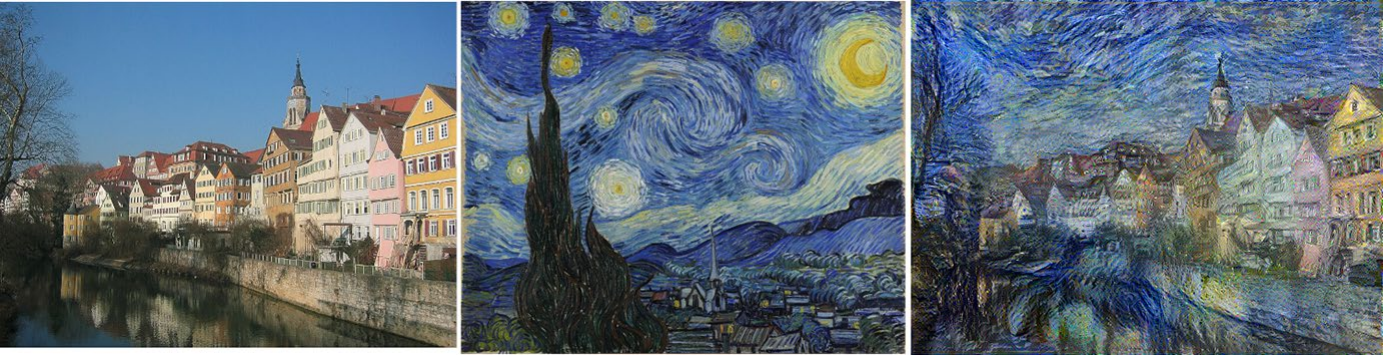
An example of artistic style transfer. Source images: Tuebingen (Andreas Praefcke, [GFDL or CC BY 3.0], Wikimedia Commons) and Vincent van Gogh’s ‘Starry Night’ (Public Domain). Final stylized image created using neural style transfer, as detailed in the TensorFlow tutorial (https://colab.research.google.com/github/tensorflow/docs/blob/master/site/en/tutorials/generative/style_transfer.ipynb#scrollTo=6msVLevwcRhm)

**Fig. 2:**
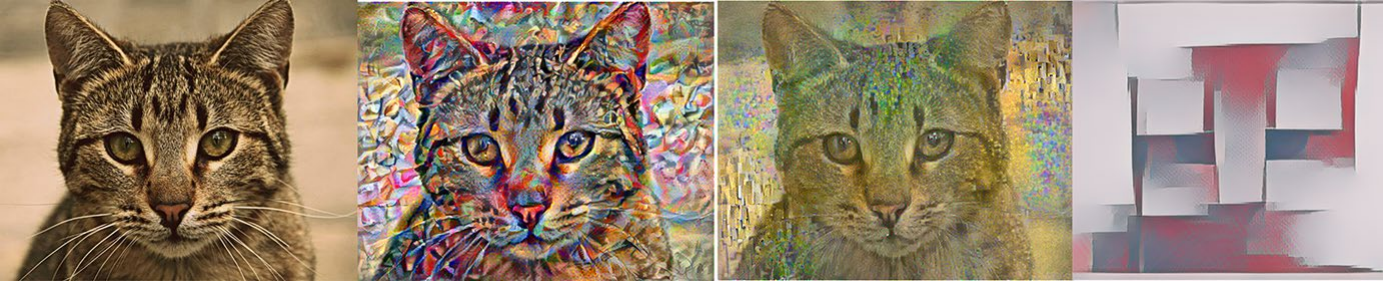
An example of artistic style transfer using different artitis styles: Vassily Kandinsky’s - Composition 7; Gustav Klimt - Thekiss; Piet Mondrian - Composición de colores nº 1 con rojo y azul. Source images: Cat (Adlum Ramadani, [GFDL or CC BY 3.0], Wikimedia Commons). Final stylized image created using neural style transfer, as detailed in the TensorFlow tutorial (https://colab.research.google.com/github/tensorflow/docs/blob/master/site/en/tutorials/generative/style_transfer.ipynb)

This model allows stimulating the production of new results from the transfer of style between images by applying artistic styles of paintings to photographs, (Gatys et al. 2015a) como se observa en la Fig. 1 y Fig. 2.

Researchers have used CNNs to transfer artistic styles to images, analyzing both the differences and similarities between artistic and non-artistic images, and identifying common properties of visual art (Simon et al. 2014). To capture more complex aspects of the original artistic style, researchers are exploring various approaches. These include modifying how the artistic style is represented, gathering more detailed information about the style, and applying more precise quality constraints to the style transfer results (Novak and Nikulin 2016).

Some studies have used CNN architecture for the analysis of image emotions, for instance, to label the emotional valence of Flickr images, (You et al. 2017); another study used the RMTHG (Rolling Mulli-Task Hypergraph) learning-based method and the combination of algorithms that propose a large-scale classification of Flickr image data for experimentation and testing, personalized emotion perception of images was managed using factors such as visual content, social context, temporal evolution and location (Zhao and Yao, 2016).

At determining the impact of specific regions of a visual image with emotional content, the researchers developed two models from CNN: one oriented to attention mechanisms that allowed an emotion classifier to be built in these regions (You et al. 2017). The other experiment applied CNS (central nervous system) deep metric learning that enabled pattern recognition and analysis for the retrieval and classification of affective images through an emotion vector using texture information (Yang et al. 2018).

The study on photographs and human emotions shows that different people have different emotional reactions to the same image and the person can present multiple emotional reactions to an image, by using CNN that adjust the color tone and texture it is possible to change the evoked emotion of an image (Peng 2017).

It is required to consider and understand human subjectivity and the context in which emotion is perceived, as a result essential relationships are established between computational image analysis and psychology, aesthetics in visual art and photography (Perrett et al. 1994; Zhang et al. 2013; Sartori et al. 2015; Peng 2017; Yang et al. 2018). CNN-based emotion classification methods have demonstrated superior performance in predicting emotions from images posted on the Internet, compared to traditional methods such as natural language processing emotional label classification for images (Cambria et al. 2017; Ahsan et al. 2017; Oliveira et al. 2018; Bonasoli et al. 2018; Xiong et al. 2019; Yadav and Vishwakarma 2020; Alexandru et al. 2022).

## Justification

The rapid spread of news with emotionally powerful images on social media, combined with a lack of verification and filtering, makes it difficult for people to identify manipulated images. Viewers often rely on the source, the platform’s interface, their own biases, and accompanying text to judge an image’s authenticity, rather than on the image itself. While some corrections are made, they are infrequent and inconsistent, failing to fully address the widespread misinterpretation of visual information online. (Metzger and Flanagin 2015; Allcott and Gentzkow 2017; Kasra et al. 2018; Meinert et al. 2018; Walter and Tukachinsky 2020).

Neither human moderation nor artificial intelligence are capable of controlling the spread of harmful content from strong images published in Internet news, when toxic online communities on conventional platforms face moderation measures, such as bans, they can migrate to other platforms (Farid 2021; Ribeiro et al. 2021). These aspects raise different questions regarding the emotional effects such as fear, disgust and surprise of strong images published in the news on social networks converted into an environment influenced by emotional distress, distrust, uncertainty, misinformation and deception.

To address the illegal and harmful content of photographs published in social media news, additional technologies are required that filter this content to a more manageable volume that mitigates the psychological effects presented. An alternative is the application of CNN that decode an original photograph, transferring it to an artistic style (Gatys et al. 2015a, b). Studies related to psychology of digital filters examine how photographic filters affect perception and increase viewers’ interpretation levels by perceiving images in a more abstract and interpretive way (Peng and Iii 2018). Two studies (Karunakaran and Ramakrishan 2019; Das et al. 2020) explored how filtering images could lessen their emotional impact on content moderators. They used grayscale and blurring (low-pass) filters and observed a mitigating effect. We anticipate that our more sophisticated “art filter” will provide comparable results. This kind of filters.

significantly changes the look of photography by providing users with convenient and fun ways to distance their images from reality by producing an artistic experience and a new interpretation of the perceived image content (Trope and Liberman 2010). In this context, applying artistic styles to images using Convolutional Neural Networks (CNNs) could offer a way to lessen the emotional impact of powerful visuals on the web. We propose the term “filter inoculation” for this process, suggesting that it might help individuals cope with potentially stressful images or better evaluate the credibility of online information.

## Objective

The objective of this research is to study how the application of artistic filters CNN are an alternative to mitigate the emotional response of strong photographs published on the Internet, for which we propose to evaluate the emotional response of images from the IAPS (International Affective Picture System) of the that we already know their emotional assessment in valence or arousal, after having been inoculated with a CNN filter of Van Gogh’s artistic style.

## Hypothesis

The perception of emotional valence will change with the application of the CNN art filter, so it is expected that inoculated versions of the IAPS images will be judged as causing less emotional arousal and less unpleasant valence.

## Method

The present research aims to use the artificial system based on CNN. It is a filter that captures paintings, identifies their characteristics and apply the artistic style to a photographic image, allowing artistic images of high perceptual quality, consisting of layers of specialized units that are used to process visual information in a hierarchical manner, each of the layers produces different shape maps that are the filtered input image. This method synthesizes the image through the application of Van Gogh’s artistic style, independently captures the content of each photographic image selected from the IAPS, identifies its characteristics and the result is a painting with textures. It is presented by designing new stimuli that introduce two independent perceptually significant sources of variation: the appearance and content of an image (Gatys et al. 2015a, b). His work used a CNN that has the ability to separate the style and content of paintings following the method of stylization proposed by Gatys et al. (2015a, b). As a biologically-inspired model of vision, CNN has demonstrated near-human performance in recognizing objects and faces. Its works examined artworks as a unit constructed by their formal elements of the artwork is regarded as a more defined procedure. This method employs a pre-trained Convolutional Neural Network (CNN), such as VGG (Visual Geometry Group), typically trained on a large image dataset. The network’s hierarchical structure extracts feature maps from different layers, each representing different aspects of the image. A randomly initialized image is then iteratively modified to combine the content of the original image with the desired style, guided by these feature maps. After several iterations, the resulting image retains the original content while adopting the style of the reference artwork. This process, based on the Gatys et al. (2015a, b) method, one of the first CNN-based approaches to image stylization, was selected because it effectively addresses our need to generate stylistic versions of the IAPS images.

We chose Vincent van Gogh’s post-impressionist style for two reasons. Firstly, his paintings, despite their subjective nature, clearly depict physical objects. Secondly, Van Gogh’s prolific body of work provides a large and varied dataset, which is ideal for training the CNN. The Vincent Van Gogh artistic style is characterized by visible, broad, repetitive brush strokes and had the ability to represent objects with a certain level of abstraction. In his paintings, colors were used to capture the mood instead to be used realistically, he often deliberately restricted his palette to a few colors; his paintings include portraits, self-portraits, landscapes and still life (Li et al. 2012). Furthermore, this style is widely used in the CNN field.

## Participants

A total of 140 people participated in the study aged between 23 and 25 years, 70 were men and 70 women. This number of participants is compatible with an estimated sample size considering a 2-way ANOVA (2 × 4), a medium effect size and a power of 0.8.

For the experiment, the independent variable (VI) was the emotional stimuli OI and CNN, the dependent variables (DV) were the assessment of the valence and activation dimensions of the OI and the CNN using the 9-point SAM scale. The valence dimension corresponds to the level of pleasant or unpleasant that the image makes you feel; 9 means high level, 5 intermediate point and one lower level. The second dimension corresponded to the activation that goes from the state of alert to the state of calm, 1 means very calm, 5 neutral and 9 very alert.

The independent variables were distributed in four blocks selected under four conditions: Blocks 1 High Activation—High Valence, 2 Low Valence—Low Activation, 3 High Activation—Low Valence and 4 Low Activation, High Valence, following the IAPS affective ratings (lang et al. 2008). The participant results, in terms of valence and activation were compared with the original values of valence and activation reported by lang et al. (2008)

## Materials

A total of 64 images were selected from the IAPS bank using their valence and activation as a criterion following the IAPS affective ratings (lang et al. 2008) (see appendix 1) and distributed in four conditions with the same number of images: 1st high activation—high valence (see appendix 2), 2nd low valence—low activation (see appendix 3), 3rd high activation—low valence (see appendix 4) and 4th low activation—high valence (see appendix 5). High-activation images were considered to be those with an average activation between 5.76 and 7.35 (values higher than the third quartile), and those with an average of low activation between 1.72 and 3.97 (values below the first quartile). In terms of valence, mean values between 6.75 and 8.34 (values above the third quartile) were considered high, and mean values between 1.31 and 3.53 (values below the first quartile) were considered low valence. These 64 images were later inoculated into CNN in such a way that each block ended up consisting of 16 OIs and 16 CNN for a total of 128 stimuli. During the filter inoculation process, we discarded any CNN-generated images that significantly deviated from the originals. Images with extraneous details not part of the artistic style were also rejected. This was done to ensure that the context of the original image was preserved.

The OpenSesame 3 software was used, it is an program that allows you to create the experiment, generates graphic experiences, through the tutorial the experiment will be structured step by step. to control the presentation of stimuli and record the participants’ responses, (Mathôt et al. 2012). The task was presented on a SAMSUNG 8 GB DDR4 memory computer, 1 TB 5400 RPM hard drive with a 1366 X 768 resolution display. (Mathôt et al. 2012). The task was presented on a SAMSUNG 8 GB DDR4 memory computer, 1 TB 5400 RPM hard drive with a 1366 X 768 resolution display.

**Fig. 3:**
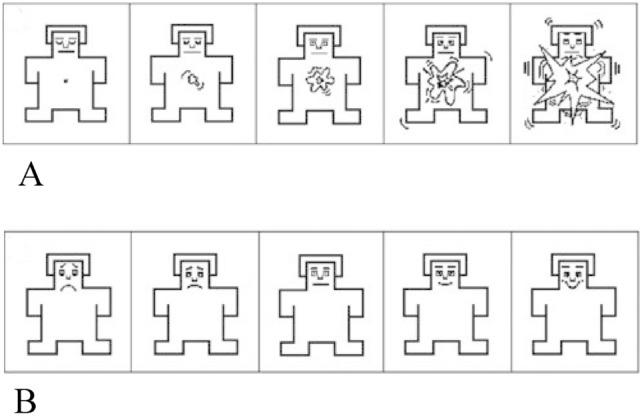
SAM scales of **A** arousal and **B** valences used to record participants’ emotional response. Source: (Bradley and Lang 1994)

To record the emotional response, the SAM scale (Self-Assessment Maniki: Self-Assessment Scale with Mannequins) (Bradley and Lang 1994; Bradley and Lang 1994) was used, in an iconographic format that represents as directly as possible the emotional reactions of those evaluated in each of the two dimensions (see Fig. 3).

## Design

**Fig. 4:**
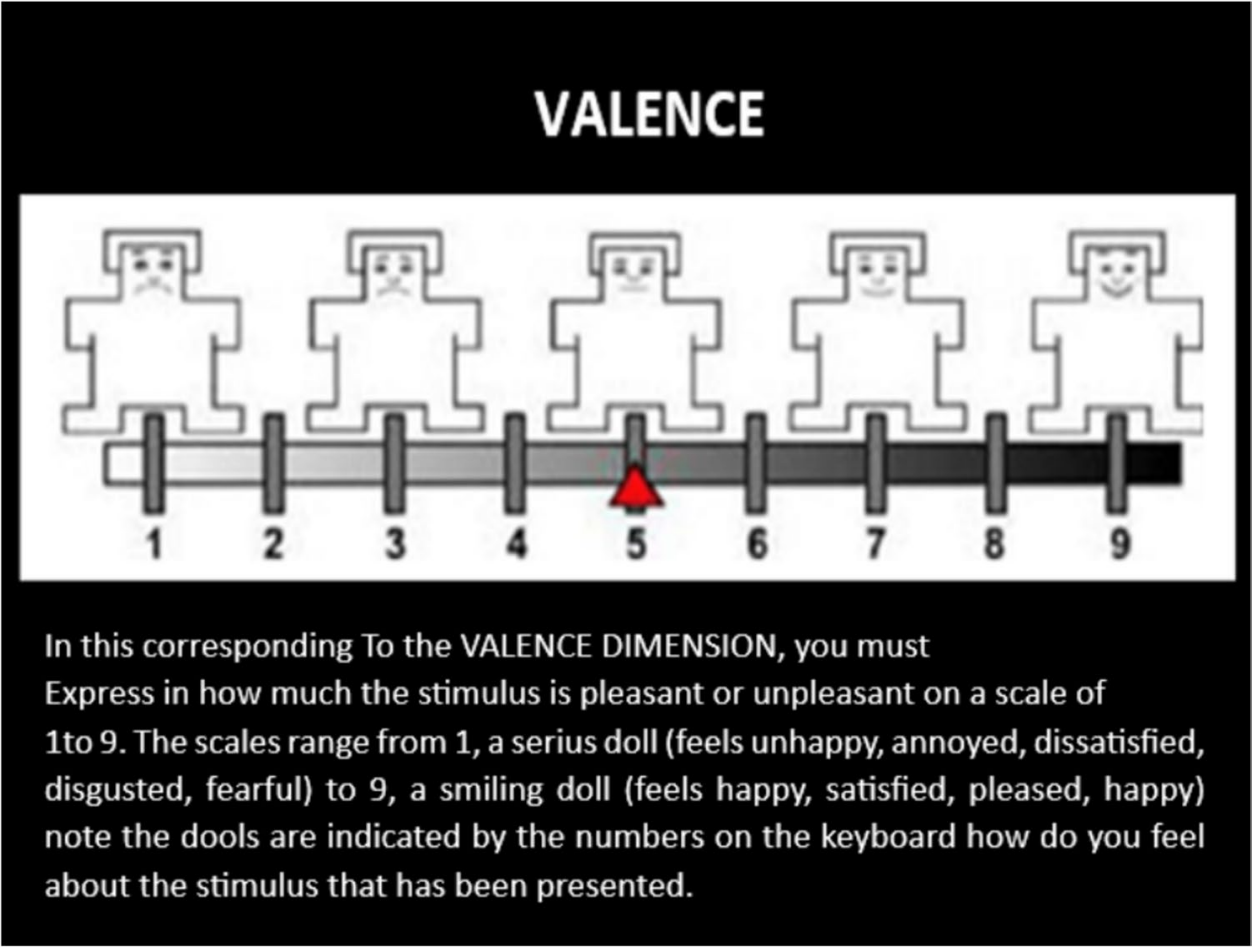
Instructions on how to use the SAM valence scale. Source: (Bradley and Lang 1994)

**Fig. 5:**
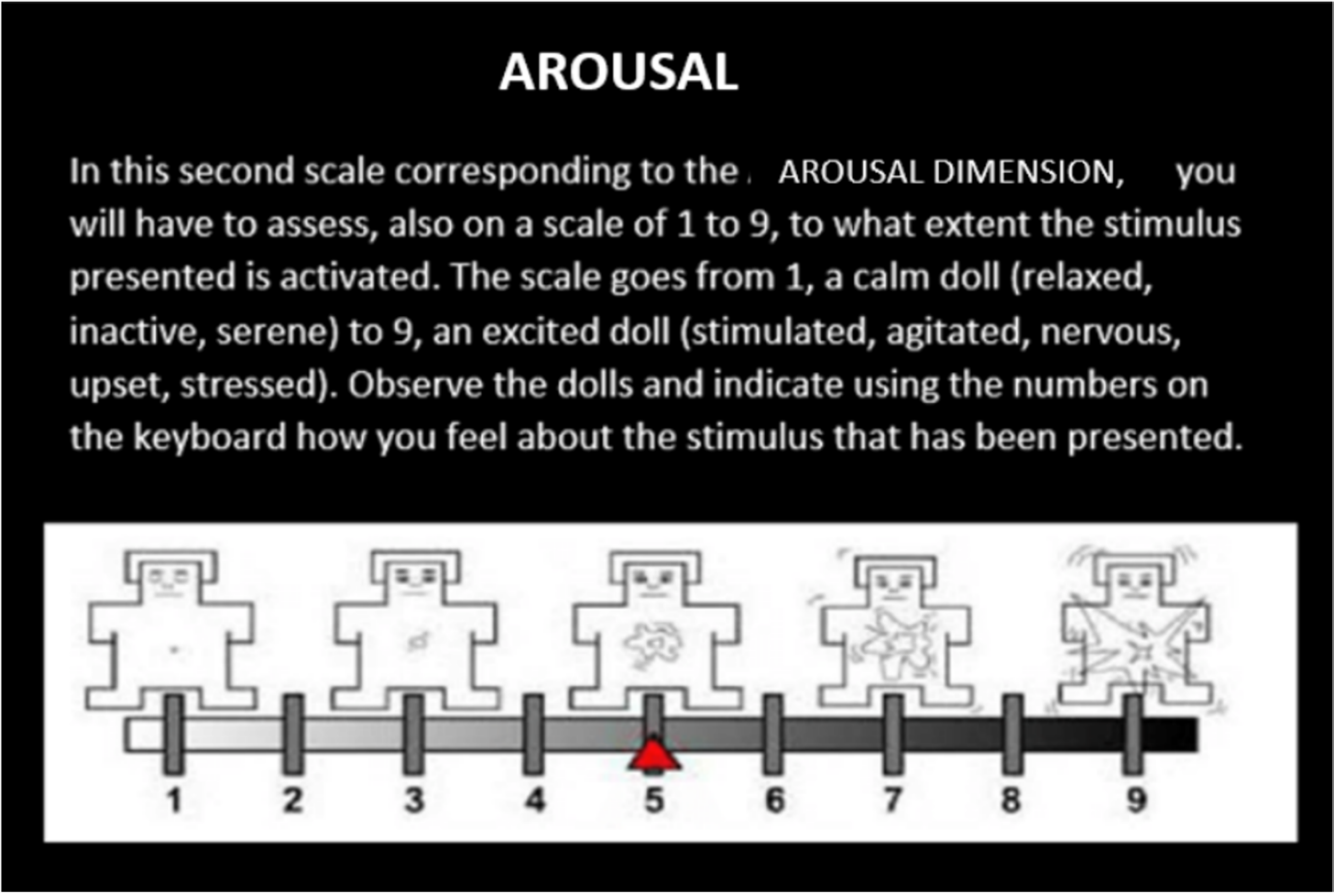
Screen for instructions on how to use the arousal SAM scale. tags. Source: (Bradley and Lang 1994)

The experimental task began with the presentation of the instructions on the screen explaining the task and how the SAM valence scales should be used as indicated in Fig. 3 and activation. To do this, the images of each of the SAM scales appeared on the screen with nine numbers aligned at the bottom of the image, indicating to the participant that they had to type a number between 1 and 9 on the keyboard. Low numbers indicate little pleasure/arousal, high numbers indicate a lot of pleasure/arousal, and the number 5 means neutral or intermediate, as indicated in Figs. 4 and 5.

The participants are informed about the development of the experiment related to the evaluation of different emotional images, according to each of the four blocks of images that are randomly presented, each block will have two events: event one corresponds to the presentation of the OI image to be evaluated, event two of each block corresponds to the evaluation of the image inoculated through artistic filters.

Participants sat about 60 cm in front of the monitor and only began the experimental task after having accepted the term of free and informed consent. Once the doubts were clarified, the experimental blocks began, which consisted of the presentation of each set of OIs and their respective CNN version, accompanied by the respective SAM scales. The presentation of each set of images on the monitor begins with a fixation point always followed by the OI and the respective screens for the evaluation of arousal and valence, to subsequently present the CNN image with the respective SAM scales.

We chose to presented the OR version of the image always before the inoculated one, because we want to evaluate how much the level of activation changed when an inoculated version of the same image is presented then immediately. On the hypothesis that the inoculation on the image that already know could cause a change in the emotional response.

**Fig. 6:**
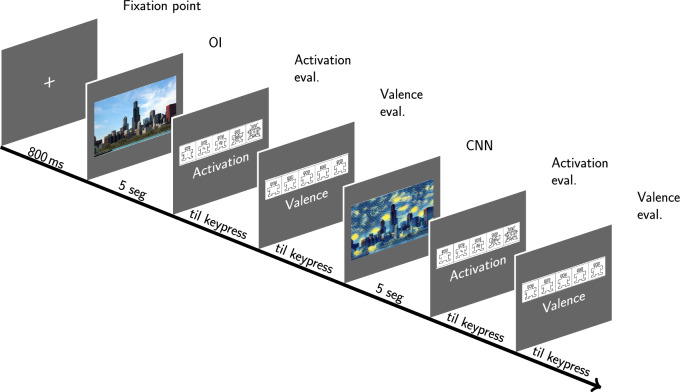
Diagram of the presentation of each set of OI and CNN images and the respective valence and activation evaluations caused by them. Source: The author

The presentation of the images, as well as the scales, had a predetermined time limit (5 s for the OI and their CNN versions and free time to respond to each of the SAM scales), and each valence-combination block arousal occurred randomly. The task ended when participants finished evaluating all 128 images, as shown in Fig. 6.

## Data analysis

For data analysis, repeated measurements (2 × 4) were performed with two intra-subject factors: the image factor with two levels, real and CNN, and the block factor with 4 levels, high activation-high valence; low valence and activation; low activation and high valence; Low activation and high valence. The Anova was preceded by normality tests and subsequently post-hoc tests of multiple comparisons were performed using a Bonferroni fit. In all cases, a p-value of less than 0.05 was significant.

## Results

The comparison of the results of the assessments was carried out using the experimental design model, in which the blocks, the dimensions (valence and arousal) and the OIs and CNNs are compared, the results allow us to know if the OI represents the same emotional experience compared to the inoculated CNN image. In order to check if there are differences with respect to the valence and arousal dimensions of the OI and the CNNs, ANOVA was used with a significance level of 0.05, testing the hypothesis of equality in the average ratings. The results are as follows: Table 1.

**Table 1:**
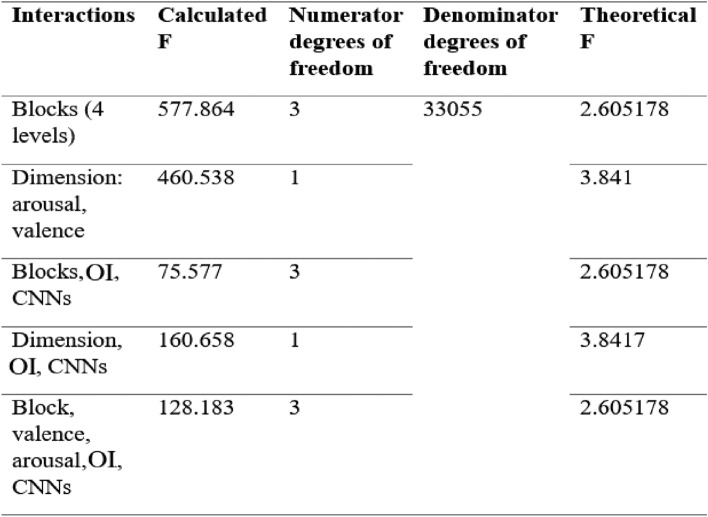
Anova analysis of the valuations of the valence and arousal dimensions of the OI and the CNNs

The average values between blocks differ Source: The author.

**Fig. 7:**
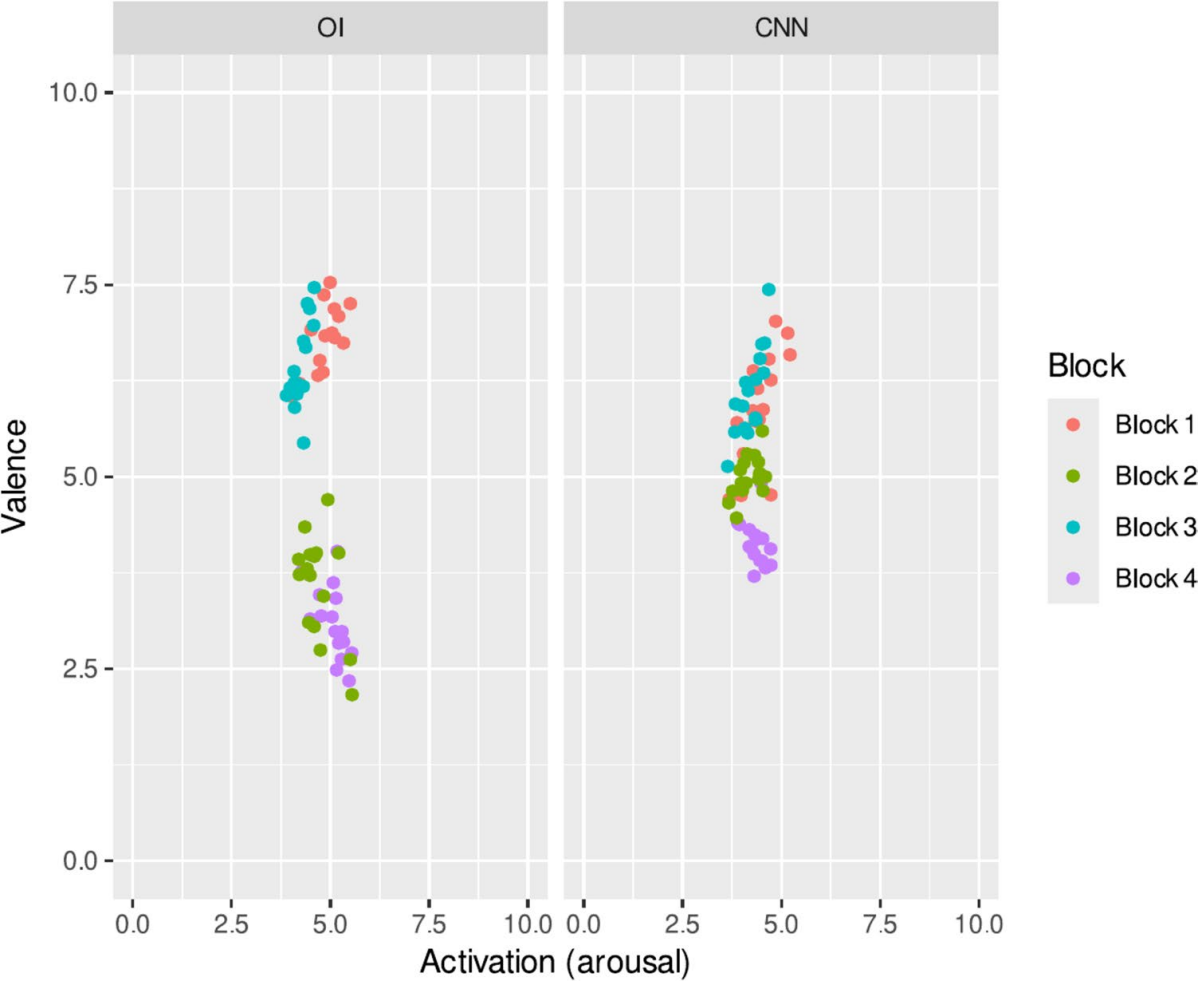
Scatter plot comparing the responses to the SAM scales for each of the images. Source: The autor

Generally speaking, manipulating images with art-style artificial neural networks decreases valence and arousal. Figure 7 presents a scatter figure that compares the values of valence and arousal attributed to both the OIs and the inoculated images and in which it is possible to observe that the arousal in general decreased, especially for block 4. Something similar occurred with the valence being concentrated around the value of 5, indicating that the inoculated images were perceived as less positive or negative.

The result of the ANOVA shows that there were differences in the way participants perceived the images after inoculation with art-style artificial neural networks, both in terms of valence and arousal.

As can be seen in Table 2, the ANOVA detected significant differences related to the images, the blocks and the image-block interaction. Post-hoc tests indicated that block 4 (low arousal—high valence) was evaluated as causing significantly lower valence than that caused by the images in blocks 1 (high arousal—high valence) and 2 (low valence—low arousal). On the other hand, block 3 (high arousal—low valence) was judged to have a significantly higher valence than blocks 2 and 3 (See Table 2).

**Table 2:**
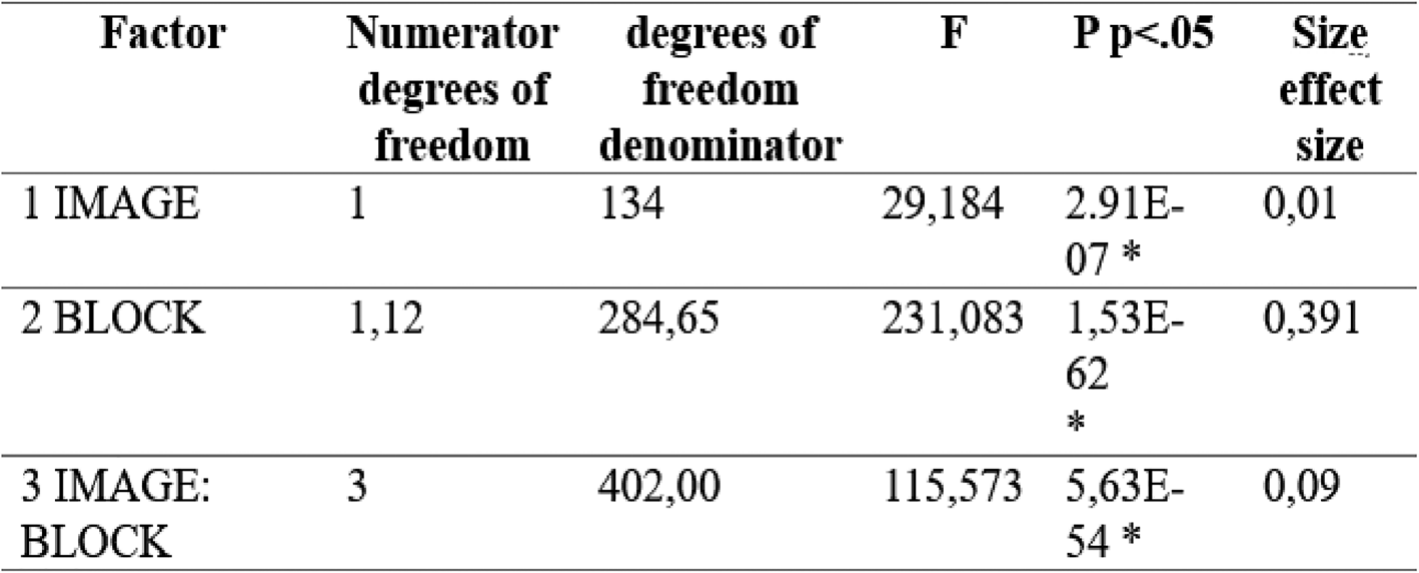
Result of the ANOVA model for valence

P values below 0.05 are considered significant. Source: The author

**Fig. 8:**
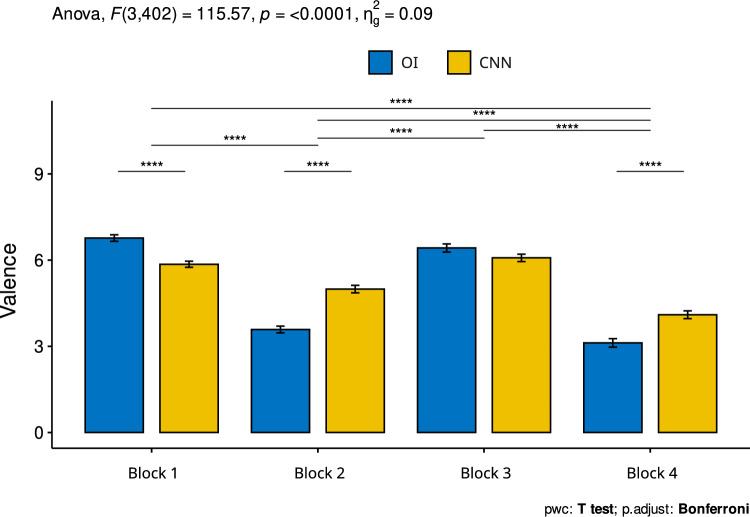
Average valence values for each of the four blocks. Error bars represent the standard error of the mean and horizontal lines represent significant differences at *** = p < 0.001. Source: The author. Block 1: high arousal-high valence; Block 2: low valence—low arousal; Block 3: high arousal—low valence and Block 4: low arousal—high valence. The horizontal lines represent significant differences between the different blocks/images

Regarding valence, significant differences were also found within blocks 1, 2 and 4, meaning that the inoculated images were considered different from those attributed to the original version. In the case of block 1 (high arousal—high valence) the inoculated images were considered to have significantly lower valence, and in the case of blocks 2 (low valence—low arousal) and 4 (low arousal—high valence) the inoculated images were judged to have significantly higher valence (see Fig. 8).

**Fig. 9:**
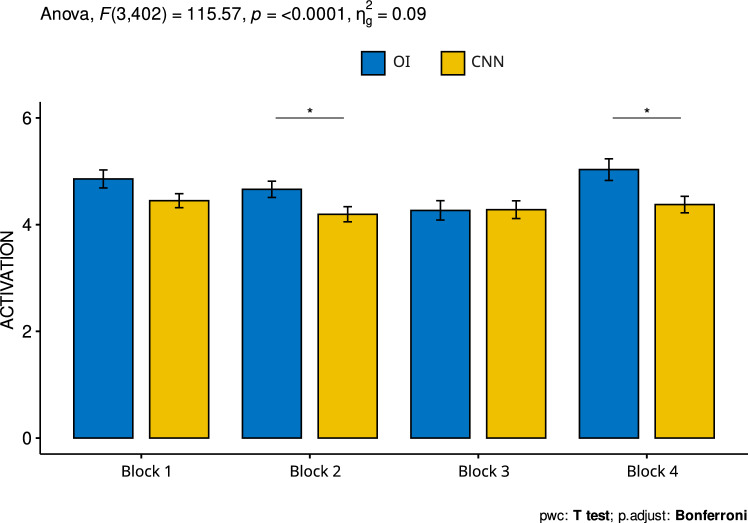
Average activation values for each of the four blocks. Error bars represent the standard error of the mean and horizontal lines represent significant differences at * = p < 0.05, Source: The author. Block 1: high arousal-high valence; Block 2: low valence—low arousal; Block 3: high arousal—low valence and Block 4: low arousal—high valence. The horizontal lines represent significant differences between the different blocks/images

Regarding arousal, Table 3 shows that the ANOVA detected significant differences related to the images and the image-block interaction. Post-hoc tests indicated that differences in the perception of arousal caused by the inoculated versions of the images within blocks 2 and 4 were judged by significantly less arousal (see Fig. 9).

**Table 3:**
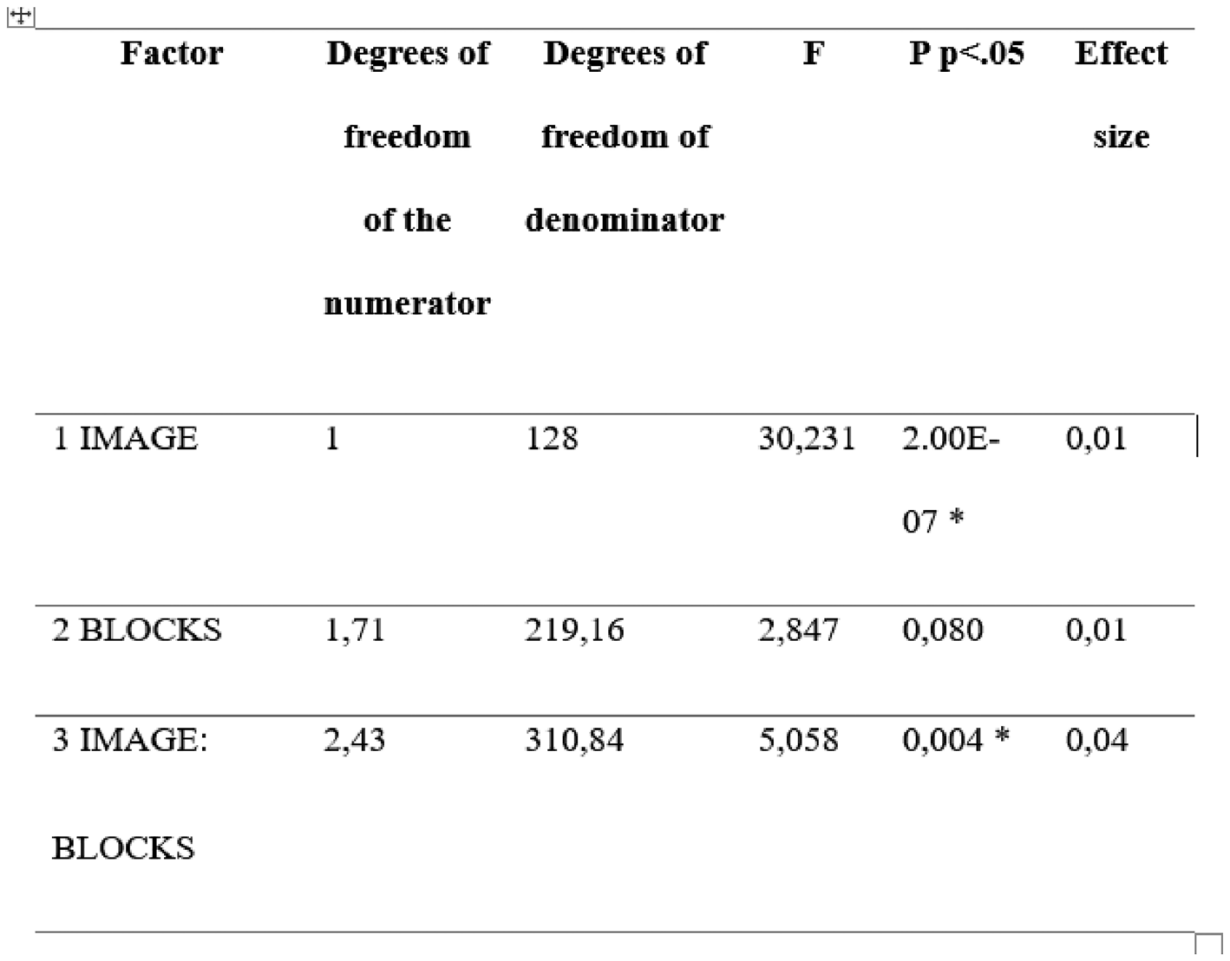
Result of the ANOVA activation model. Source: The autor

P values less than 0.05 are considered significant

## Discussion

The results show that through the evaluations of the OI and the CNNs regarding valence and arousal, no value was zero, which indicates that changes have occurred in the emotional states of the participants. The comparisons between the blocks reflect differences in the averages: block 1 presents the most significant changes in emotional states with more pleasant valence and more arousing pleasant (Bakhshi et al. 2015; Chandler and Livingston 2016; Murrin 2017); block 3 presents trending changes to more pleasantness and less tension (Peng and Iii 2018), with respect to block 2 and 4, similar trends are observed, although with different numerical evaluations in the milder behavioral changes with respect to the valences with less pleasant and less arousing pleasant experiences (Ou et al. 2004).

The averages of the comparisons of the valence and arousal dimensions of the OI and the CNN allow us to show that there are differences in the emotional states, the most significant changes are observed in the valence, where the stimuli produce alterations in the moods with a tendency towards pleasant effects (Murrin 2017); arousal reflects less significant variations with changes in the emotional experience towards calm, the evaluation carried out is a basic aspect of people that allows conclusive distinctions to be made characterizing their experiences in different types of emotions such as pleasure or displeasure (Zhao et al. 2021).

The interactions of the 4 blocks and dimensions valence and activation reflect differences, the most significant are obtained from the interaction between block 1 and the valence similar to block 3 and valence with a tendency towards changes related to pleasant emotional experiences, the interaction between block 4 and activation is similar to the interaction between block 1 and activation reflecting changes towards emotional states with a tendency to calm (Zhao et al. 2017).

The averages in the interactions between blocks and images are different, blocks 1 and 3 reflect representative changes in the evaluations of the OI and the CNN, while the evaluations of blocks 3 and 4 are similar in the OI and the CNN, (Gatys, Ecker and Bethge 2015c). The averages relating to the interactions between the dimensions, the OI and the CNNs reflect differences in the evaluations, the CNN and the valence present the most significant changes, meaning that the effects produced by the CNN are more pleasant, with respect to activation, OIs tend to produce effects of greater displeasure and tension, while CNNs present reactions that tend toward calm. (Davitz 1964; Osgood et al. 1957; Mehrabian and Russell 1974; Joshi et al. 2011).

## Conclusion

In summary, the present research demonstrated how the application of artistic filters with CNNs are an alternative to mitigate the emotional response to strong photographs published on the Internet. Using the neural network-based architecture of CNNs they inoculated a OI through artistic filters and participants separately evaluated OIs and inoculated images using the dimensions of emotional valence (unpleasant to pleasant) and arousal (calming to exciting). The ratings show that there were differences in how participants perceived the images; After inoculation with CNN, emotional reactivity decreased, showing that the effects produced by CNNs are more pleasant with respect to the valence and with respect to the arousal of OIs. Uninoculated images tended to produce more tense effects, while images inoculated with filters made with CNN provoked reactions that tended to be calm. The changes in the most significant emotional states are observed in valence, new stimuli produce alterations with a tendency to pleasant effects. Simultaneously, these results may allow the development of another methodology that, based on different artistic filters originating from different artistic styles derived from original paintings, creates a new photographic image that can replace strong photographs published in the news on the Internet. On the other hand, although the changes reflected in arousal present less significant variations, the trend showed a change in the emotional experience towards calm, suggesting that it is possible to create unique experiences through the artistic style of an image.

In this way, the strong photographic image inoculated from the artistic filter with CNN will have an emotional value with less emotional arousal and less negative valence. The results suggest that strong photographs published in Internet news inoculated to a CNN art style can be used to mitigate users emotional response. The findings present a viable way at a theoretical level to relate emotion, photographic image and CNN artistic filters, digital technology applied to media, aesthetics and users, among others.

Our research blends psychology, art, AI, and computer science to investigate how applying artistic styles to images using CNNs can affect viewers’ emotional responses. We call this process “filter inoculation.” The primary goal is to assess the potential of this technique in various fields, such as medicine, communication, and psychotherapy, as a tool for managing reactions to emotionally powerful images. Future research will explore applying and analyzing different artistic styles, potentially mixing them, and investigating alternative inoculation methods. We also plan to broaden the research to include aesthetic emotions and other media, such as music, videos, animations, and films.

## Limitations

This study has limitations that should be acknowledged. First, the image set used, drawn from the international affective picture system (IAPS), may not be entirely equivalent to images found in news or on social media. IAPS images often depict naturalistic situations, while online images are frequently more digitally manipulated. However, the IAPS remains one of the most validated sets of images for eliciting emotional responses.

Another limitation is the use of only one artistic style: that of Vincent van Gogh. While this choice aimed for a consistent application of a style that would minimize unexpected emotional reactions due to unfamiliarity, it may have introduced a different bias. Viewers who dislike Van Gogh’s style might experience negative emotions, potentially affecting the study’s results.

## Data Availability

No datasets were generated or analysed during the current study.
